# Design and rationale of the RISC-trial: multicentre *R*CT to assess *i*mmediate discharge of *s*yncope patients admitted to the (*c*ardiac) emergency room

**DOI:** 10.1093/ehjopen/oeag078

**Published:** 2026-05-14

**Authors:** Thomas T Boel, Suzanne Y G Peeters, Anouk P van Alem, Mark J Boogers, Marianne Bootsma, Marinus A van den Dorpel, Mehran Firouzi, Jan G J Groothuis, Martin E W Hemels, Jaco H Houtgraaf, Ward P J Jansen, Jelle S Y de Jong, Monica Jongenotter, Evert C A Kaal, Egbert M Koomen, Susan E Langerak, Gideon Mairuhu, Nieke E Mullaart, Johannes B van Rees, Mariam Samim, Eric Wierda, Steven van Zanten, Arthur van Zoelen, Marit van Barreveld, Philip M Croon, Marcel G W Dijkgraaf, Jan G P Tijssen, Joris R De Groot, Frederik J de Lange

**Affiliations:** Department of Clinical and Experimental Cardiology, Heart Centre, University of Amsterdam, Amsterdam Cardiovascular Sciences, Amsterdam UMC, Meibergdreef 9, Amsterdam 1105AZ, The Netherlands; Department of Emergency Medicine, Flevo Hospital, Hospitaalweg 1, Almere 1315RA, The Netherlands; Department of Cardiology, Haaglanden Medical Center, Lijnbaan 32, The Hague 2512VA, The Netherlands; Department of Cardiology, Heart-Lung Center, Leiden University Medical Center, Albinusdreef 2, Leiden 2333ZA, The Netherlands; Willem Einthoven Center of Arrhythmia Research and Management, Department of Cardiology, Leiden University Medical Center, Albinusdreef 2, Leiden 2333ZA, The Netherlands; Department of Cardiology, Heart-Lung Center, Leiden University Medical Center, Albinusdreef 2, Leiden 2333ZA, The Netherlands; Willem Einthoven Center of Arrhythmia Research and Management, Department of Cardiology, Leiden University Medical Center, Albinusdreef 2, Leiden 2333ZA, The Netherlands; Department of Internal Medicine, Maasstad Hospital, Maasstadweg 21, Rotterdam 3079DZ, The Netherlands; Department of Cardiology, Maasstad Hospital, Maasstadweg 21, Rotterdam 3079DZ, The Netherlands; Department of Cardiology, Diakonessenhuis, Bosboomstraat 1, Utrecht 3582KE, The Netherlands; Department of Cardiology, Rijnstate Hospital, Wagnerlaan 55,. Arnhem 6815AD, The Netherlands; Department of Cardiology, Radboud University Medical Centre, Geert Grooteplein Zuid, Nijmegen 6525GA, The Netherlands; Department of Cardiology, Rijnstate Hospital, Wagnerlaan 55,. Arnhem 6815AD, The Netherlands; Department of Cardiology, Tergooi MC, Laan van Tergooi 2, Hilversum 1212VG, The Netherlands; Department of Clinical and Experimental Cardiology, Heart Centre, University of Amsterdam, Amsterdam Cardiovascular Sciences, Amsterdam UMC, Meibergdreef 9, Amsterdam 1105AZ, The Netherlands; Department of Cardiology, Rivas Hospital, Banneweg 57, Gorinchem 4204AA, The Netherlands; Department of Neurology, Maasstad Hospital, Maasstadweg 21, Rotterdam 3079DZ, The Netherlands; Department of Cardiology, Gelre Hospital, Albert Schweitzerlaan 31, Apeldoorn 7334DZ, The Netherlands; Department of Cardiology, Haaglanden Medical Center, Lijnbaan 32, The Hague 2512VA, The Netherlands; Department of Cardiology, Flevo Hospital, Hospitaalweg 1, 1315RA Almere, The Netherlands; Department of Emergency Medicine, Dijklander Hospital, Maelsonstraat 3, Hoorn 1624NP, The Netherlands; Department of Cardiology, Rijnstate Hospital, Wagnerlaan 55,. Arnhem 6815AD, The Netherlands; Department of Cardiology, Maasstad Hospital, Maasstadweg 21, Rotterdam 3079DZ, The Netherlands; Department of Cardiology, Dijklander Hospital, Maelsonstraat 3, Hoorn 1624NP, The Netherlands; Department of Clinical and Experimental Cardiology, Heart Centre, University of Amsterdam, Amsterdam Cardiovascular Sciences, Amsterdam UMC, Meibergdreef 9, Amsterdam 1105AZ, The Netherlands; Department of Cardiology, Reinier de Graaf Hospital, Reinier de Graafweg 5 Delft 2625AD, The Netherlands; Department of Cardiology, Rivas Hospital, Banneweg 57, Gorinchem 4204AA, The Netherlands; Department of Epidemiology and Data Science, Amsterdam UMC, Meibergdreef 9 Amsterdam 1105AZ, The Netherlands; Methodology, Amsterdam Public Health, University of Amsterdam, Meibergdreef 9, Amsterdam 1105AZ, The Netherlands; Section of Cardiovascular Medicine, Department of Internal Medicine, Yale School of Medicine, 333 Cedar St, New Haven, CT 06510,USA; Department of Epidemiology and Data Science, Amsterdam UMC, Meibergdreef 9 Amsterdam 1105AZ, The Netherlands; Methodology, Amsterdam Public Health, University of Amsterdam, Meibergdreef 9, Amsterdam 1105AZ, The Netherlands; Department of Epidemiology and Data Science, Amsterdam UMC, Meibergdreef 9 Amsterdam 1105AZ, The Netherlands; Methodology, Amsterdam Public Health, University of Amsterdam, Meibergdreef 9, Amsterdam 1105AZ, The Netherlands; Department of Clinical and Experimental Cardiology, Heart Centre, University of Amsterdam, Amsterdam Cardiovascular Sciences, Amsterdam UMC, Meibergdreef 9, Amsterdam 1105AZ, The Netherlands; Department of Clinical and Experimental Cardiology, Heart Centre, University of Amsterdam, Amsterdam Cardiovascular Sciences, Amsterdam UMC, Meibergdreef 9, Amsterdam 1105AZ, The Netherlands

**Keywords:** Syncope, Risk stratification, Acute care, Health care evaluation

## Abstract

**Aims:**

Syncope is responsible for 1–3% of emergency department presentations. The European Society of Cardiology guidelines on syncope recommend a structured initial evaluation to diagnose syncope patients. If the cause of the syncopal event remains unexplained, risk-stratification is applied to distinguish between patients at high risk of serious cardiovascular events and low-risk patients, who can be safely discharged. However, there is a group of patients in whom this risk can currently not be adequately determined, hereafter called intermediate risk. In clinical practice, these patients are either immediately discharged or admitted for 24 h in-hospital cardiac rhythm monitoring. Due to the high cost of admission, immediate discharge could lower healthcare costs, hospital bed utilization, and patient burden. To our knowledge, there is no sufficient scientific basis for the superiority of either. The RISC trial aims to resolve this knowledge gap.

**Study Design:**

The RISC trial is an investigator-initiated multicentre prospective randomized controlled trial. A total of 640 predefined low- and intermediate-risk syncope patients are randomized to either immediate discharge or discharge after 24 h in-hospital rhythm monitoring (1:1). The trial is powered to claim non-inferiority of immediate discharge with respect to (non-)fatal composite safety endpoints, which entail arrhythmic disorders, syncope recurrence, and (cardiovascular)death. Furthermore, healthcare costs and quality of life will be evaluated.

**Conclusion:**

The RISC trial is a multicentre RCT that aims to gather scientific evidence for immediate discharge of the ESC guideline–based pre-defined low- and intermediate-risk syncope patients in the emergency department. Primary endpoints entail cardiovascular events such as arrhythmias, syncope, and death. This trial is registered at ClinicalTrials.gov with trial-ID: NCT06472375.

## Graphical Abstract

**Graphical Abstract:**
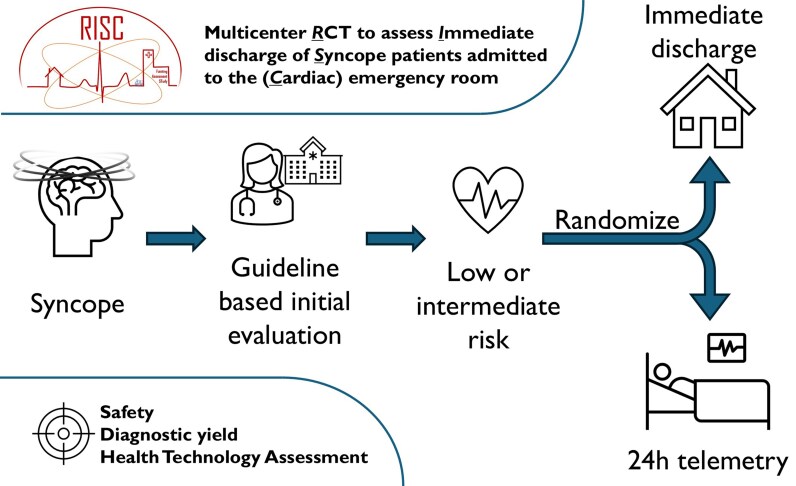

## Background

Syncope is defined as a transient loss of consciousness (TLOC) due to global cerebral hypoperfusion.^1^ Each year, one in every thousand inhabitants presents in the (cardiac) emergency department ((C)ED) with syncope,^2^ accounting for 1–3% of emergency department presentations.^3^

Syncope is a symptom with a broad differential diagnosis, spanning across the lines of medical specialties (cardiology, neurology, internal medicine, geriatrics, emergency medicine, and primary care). Additionally, causes range from benign to life-threatening. The combination of a diverse differential diagnosis spanning multiple specialties, mostly based on circumstantial evidence to establish a diagnosis on, and time constraints in emergency departments makes the evaluation of syncope patients challenging for most physicians.^4^

If the initial evaluation yields a certain/highly likely diagnosis, treatment is instigated based on the cause of the syncopal event. Cardiac syncope patients should be admitted for treatment and additional diagnostic evaluation, as they are at substantial risk of serious cardiovascular events. In contrast, patients with evident reflex syncope and orthostatic hypotension (autonomic syncope) can be safely discharged, as they do not benefit from hospital admission or observation for the diagnosis or treatment of the syncopal event.^1,5–9^

There is, however, a significant group in whom the origin of syncope is still unexplained after the initial evaluation. In these patients, the 2018 European Society of Cardiology (ESC) guideline recommends risk stratification.^1^ The ESC guideline provides high-risk features in the scope of history taking, physical examination, and 12-lead electrocardiogram. These risk features are used to stratify patients into low (no high-risk features) or high risk (multiple risk features) of serious cardiovascular events. In earlier studies, eventually adjudicated cardiac patients were adequately identified using the ESC-guideline based initial evaluation, exhibiting the safety of the guideline-based structured evaluation.^5,10–12^ The guideline recommends discharge of low-risk patients, which includes mostly patients with either suspected reflex syncope or suspected orthostatic hypotension (autonomic syncope), and admission of high-risk patients (suspected cardiac syncope).^1^ However, the management of patients in whom the risk is undetermined, hereafter called intermediate-risk patients, is not yet defined. Management in this group is precarious, as the high-minor risk feature (*Table 1*) often warrants suspicion of a cardiac culprit, but not enough to definitively require admission. The discrimination between admission and discharge in intermediate-risk patients is therefore subjective in nature, leading to heterogeneous treatment strategies across healthcare providers and potentially unnecessary admissions of suspected autonomic syncope patients. This is problematic as admission is the primary driver of syncope-related costs.^13^

**Table 1:** Features for risk stratification

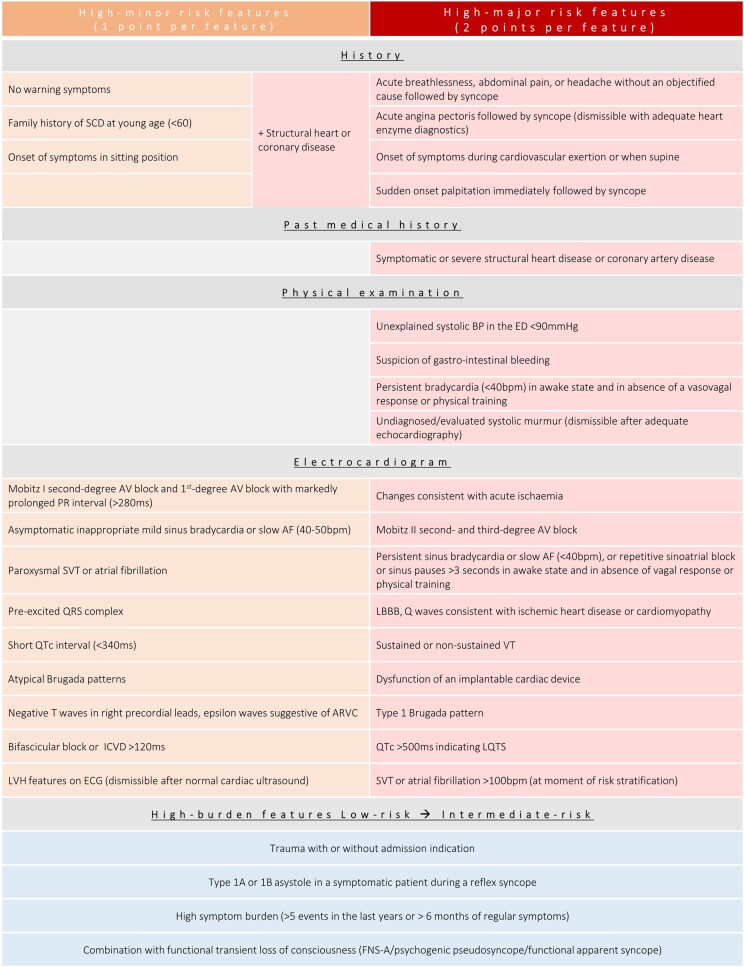

AF, Atrial fibrillation; ARVC, Arrhythmogenic Right Ventricular Cardiomyopathy; AV block, Atrio-ventricular block; BP, Blood pressure; ED Emergency department; IVCD, Intraventricular conduction disorder; LBBB, Left Bundle Branch Block; LVH, Left Ventricular Hypertrophy; LQTS, Long QT Syndrome; SCD, Sudden cardiac death; SVT, Supra ventricular tachycardia; VT, Ventricular Tachycardia

## Rationale

The current reason for the admission of syncope patients is two-fold. First, admission is considered safe because, in the case of rhythm disorders, immediate treatment can be started. Second, admission for monitoring may provide additional diagnostic value, given the chance of witnessing recurrence during the first hours after the syncopal event.^14^ Therefore, the question posed for intermediate-risk syncope patients is two-fold as well: is it safe to discharge these patients, and does admission provide a diagnostic benefit? If intermediate-risk patients can be safely discharged but might profit from rhythm observation, the solution of immediate discharge combined with immediate ambulant Holter monitoring could greatly reduce healthcare costs. Similarly, if there is no diagnostic yield, ambulant Holter monitoring could be omitted as well.

Therefore, we propose the RISC trial to investigate the safety of immediate discharge and the clinical benefit of ambulatory Holter monitoring in low- and intermediate-risk syncope patients. Furthermore, it evaluates the true healthcare cost benefit, the effect on the quality of life, the societal costs, and healthcare burden from a patient perspective. Additionally, we aim to investigate the diagnostic efficacy of immediate rhythm observation and, if apparent, the optimal duration of ambulatory Holter monitoring. The RISC trial, therefore, aims to determine whether immediate discharge with or without ambulatory Holter monitoring is safe and if it is a clinically and economically appropriate alternative to 24-h in hospital cardiac rhythm monitoring in pre-defined low- and intermediate-risk syncope patients.

## Trial design

**Figure 1:**
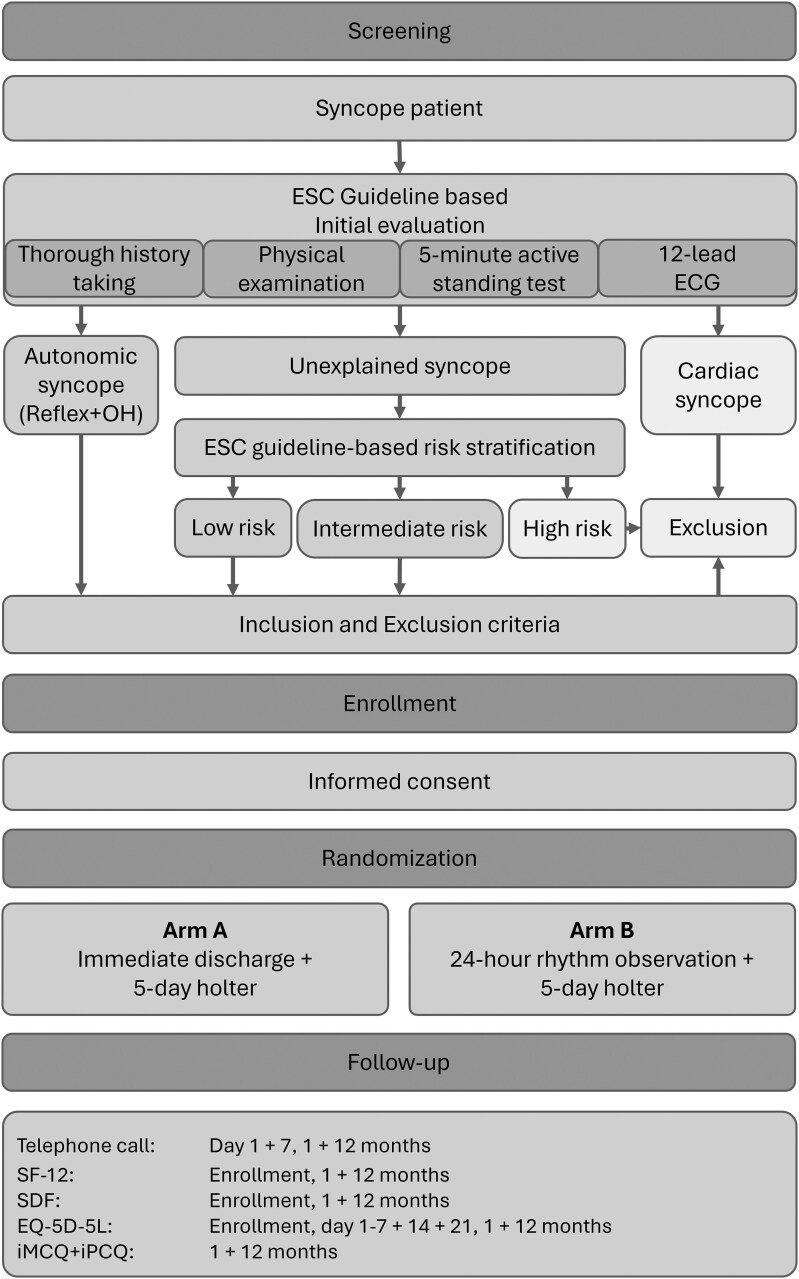
Trial design flowchart.

The RISC syncope trial is an investigator-initiated, multicentre, randomized, controlled, prospective two-arm clinical trial designed to evaluate if immediate discharge is noninferior to in-hospital 24-h cardiac rhythm observation. Patients are identified in the (cardiac) emergency departments of 10 large teaching hospitals (two academic and eight nonacademic) in the Netherlands. All syncope patients presenting to the (C)ED are analysed using the initial evaluation as described in the 2018 ESC guidelines on syncope within the first 4 h after arrival in the (C)ED. The guideline-based initial evaluation consists of thorough history taking, physical examination, active standing test, and a 12-lead electrocardiogram (*Figure 1*) and is part of standard care in the (C)ED in all participating hospitals.

### Patient selection

If the initial evaluation yields a highly likely or certain diagnosis of autonomic syncope (low-risk diagnosis), patients are screened for enrolment. The distinction between low or intermediate risk for subgroup analysis is then made using the ESC guideline–based RISC score (see next paragraph).

Patients in whom no highly likely or certain diagnosis can be established (unexplained diagnosis), ESC guideline–based RISC stratification will be performed (see next paragraph). Low- and intermediate-risk syncope patients are screened for enrolment.

Before enrolment, patients are screened using the inclusion and exclusion criteria (*Table 2*).

**Table 2:** Inclusion and exclusion criteria


*Inclusion criteria*
All low and intermediate cardiac risk syncope patients on the (C)ED
>18 years old
Able to provide informed consent
*Exclusion criteria*
Those aged <18 years Stratification as high risk
Contraindication for early discharge Certain or highly likely cardiac diagnosis Illness Trauma Social
Near-syncope
Unable to provide informed consent Unable to fill in questionnaires

### Risk stratification: ESC guideline–based RISC score

**Figure 2:**
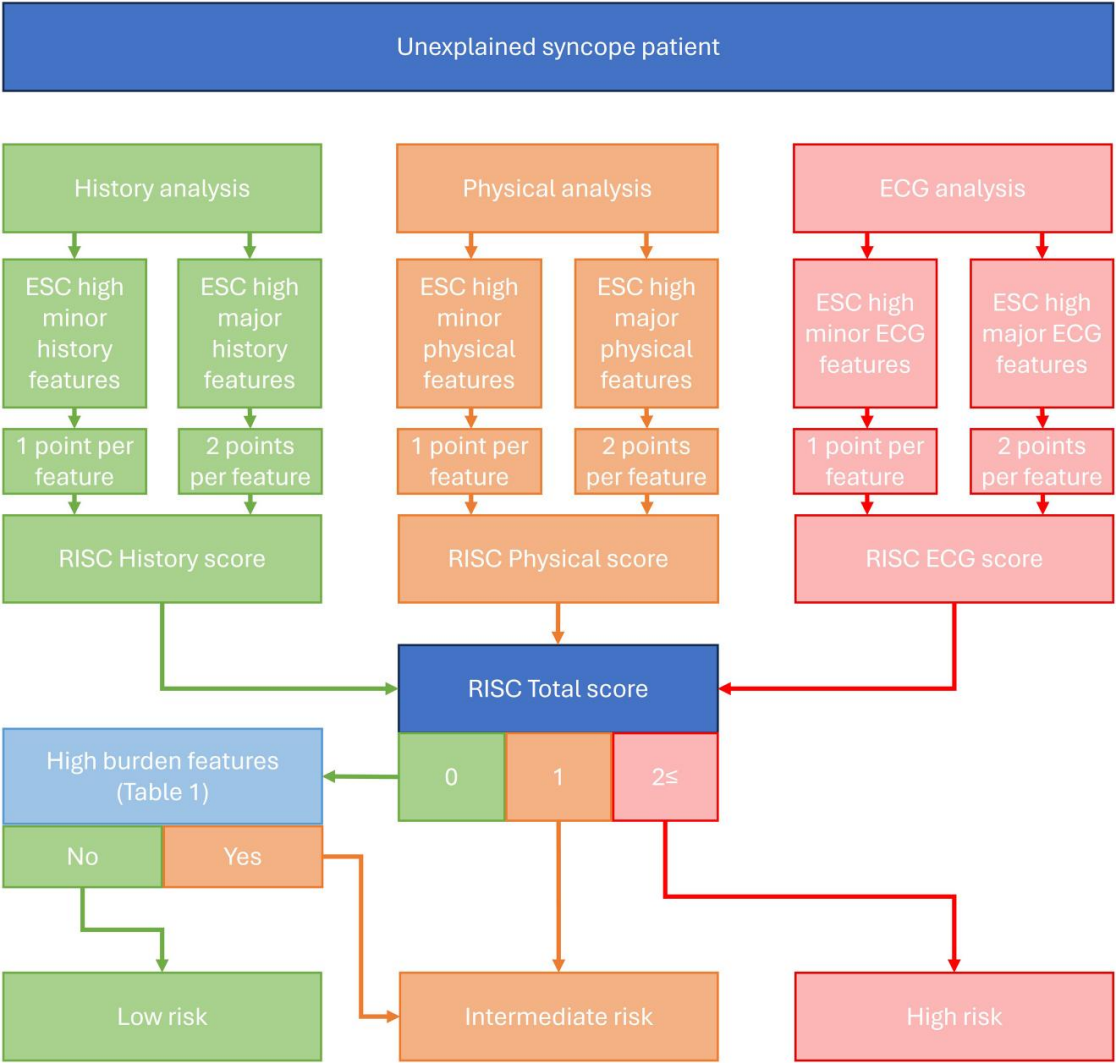
ESC-guideline based RISC-score calculation.

Patients will be risk-stratified using the pre-defined ESC guideline–based RISC score (*Figure 2*). This score is calculated by assigning 1 point to every high-minor feature and 2 points to every high-major feature (*Table 1*). All patients will then be classified in three groups: low- (0 points), intermediate- (1 point), and high-risk (2 points) of serious cardiovascular events (*Figure 2*). Additionally, patients with a low calculated risk but with ≥1 high burden feature are deemed intermediate risk; these features include trauma without admission indication, high symptom burden, documented 1A/1B asystole during a reflex syncope, or a combination with functional transient loss of consciousness (psychogenic pseudosyncope) (see *Table 1*).

### Informed consent and ethical approval

This trial adheres to the 2025 ICH-GCP guidelines.^15^ Patient information folders will be distributed to low- and intermediate-risk syncope patients identified on the (C)ED. Adamant time is provided to evaluate their participation in the trial before signing the informed consent form. This trial has been approved by the Medical Ethical Committee of the Amsterdam UMC (NL-009238) and is registered on clinicaltrials.gov (NCT06472375).

### Randomization

Upon inclusion, participants will be randomized to either the intervention arm (immediate discharge) or the comparator arm (24-h telemetry monitoring). Randomization is performed using a web-based weighted variable block design. Both study arms will receive 5-day ambulatory Holter monitoring upon randomization in order to record endpoints with the same modality. Physicians are advised to manage these patients as they would following the local protocols, with as the only confining factors being the minimal 24-h observation period and cardiac rhythm observation.

### Baseline characteristics

For comparison of the groups, baseline characteristics will be collected; these entail the following: age, height, weight, country of birth, number of TLOCs within the last year, smoking, alcohol use, drug use, medication use, frailty, comorbidities, most probable diagnosis, and low-risk, high-minor, and high-major criteria.^1^

### Questionnaires and telephone consults

Patients in both study arms were asked to complete questionnaires assessing syncope recurrence, quality of life (SF12, SDF, and EQ-5D-5L), and societal costs (iMCQ and iPCQ). Questionnaires were administered during the first week, at weeks 2, 3, and 4, and at 1 year (Appendix A). Participants are contacted by phone at day 1, day 7, 1 month, and 1 year after enrolment to verify the endpoints (Appendix A).

### Holter monitoring

Immediately after randomization, both treatment arms will receive 5-day ambulatory Holter monitoring using the Philips ePatch®.^16^ Patients are required to wear the Holter for a minimum of 24 h with sufficient signal. Holter patches are reapplied when they are not securely fixated during the first 24 h to ensure complete registration in the first 24 h for the primary endpoint analysis. After the initial 24-h period, Holter patches are not reapplied. All Holter recordings are analysed using the Cardiologs Holter analysis platform by an experienced clinician, a second expert will perform an independent quality control review.^17^ All pre-defined rhythm disturbances will be reported (Appendix C), even if they are not part of the endpoints. In the event of clinically relevant findings, treating physicians are notified immediately. The ambulatory Holters are not monitored in real time, as not to affect healthcare costs. Treating physicians will not have standard access to the full Holter report; the Holter data are available on request, allowing researchers to assess the potential diagnostic yield of the Holter monitoring.

### Endpoints

The primary safety endpoint consists of the composite of arrhythmia disorders (supraventricular tachycardia, sinus pauses or bradycardia, and ventricular tachycardia), pre-defined conduction disorders (second- or third-degree AV block), syncope recurrence, unexplained falls with injury, all-cause death, and cardiovascular death (Appendix B) at 24 h after randomization. All primary endpoints will be evaluated at 24 h. Subgroup analysis will be performed on the low-risk and intermediate-risk–only cohorts.

Secondary endpoints include clinically relevant diagnostic yield of Holter monitoring, total admission costs, number of syncope-related tests and consultations after 1 month, duration of in-hospital stay after randomization for both treatment arms (superiority), and differences in proportion of correct diagnoses in both arms. In addition, quality of life and societal costs are assessed using telephone consults and standardized questionnaires of the SF12, SDF, EQ-5D-5L, and study-adapted iMCQ and iPCQ (Appendix A). Although quality of life (measured as QALYs) may not differ due to the risk of the population closely resembling a normal population, there might be a difference in perceived safety and understanding affecting the quality of life indirectly.

All primary endpoint definitions are described in Appendix B. A critical event committee centrally reviews all events that are potential endpoints.

### Statistical considerations

Immediate discharge (Intervention) is compared with discharge after 24-h in-hospital observation with rhythm monitoring (Comparator). We chose a prospective analysis due to the patient and physician bias, which would be inherent to a retrospective design. By application of 5-day ambulant Holter monitoring for all participants, we are able to compare all (non-)fatal events in both arms for which admission is strictly not mandatory (e.g. nonrhythm issues and/or later timing of nonfatal diagnoses). Based on studies on low- and intermediate-risk syncope patients in the setting of ED and in line with data from our own institute, we anticipate an event rate of 18% in both arms.^14,18–33^ By noninferiority, we aim to show that immediate discharge of these low-/intermediate-risk syncope patients is feasible and safe. With a noninferiority limit of 9% (for the difference in event rates, 50% of 18%), inclusion of a total of 640 patients with a one-sided type-1 error of 0025 provides the trial with 80% power (10% loss included). In the RISC trial, the noninferiority margin for the risk difference was set at 9% against an expected event rate of 18%, or half of the expected event rate. This margin is equivalent to a noninferiority margin of 1.50 for relative risk. The RISC trial’s noninferiority boundary is consistent with choices made for major landmark RCTs that influenced current guidelines (examples of RCTs are available upon request). In general, the choice of the noninferiority boundary has a major impact on the sample size needed for adequate power. A stricter noninferiority boundary would necessitate an impractically large sample size. For example, a noninferiority margin of 6% against an expected event rate of 18%, equivalent to a relative risk margin of 1.33, would more than double the sample size to 2 × 644. For the inclusion rate, based on the reference areas of the participating hospitals, the annual admissions of syncope on the emergency department, this will result in ≈ 50 eligible patients per year per centre.

### Data analysis and bias

The main analysis is designed to evaluate whether immediate discharge (investigational treatment strategy) is noninferior to 24-h admission with continuous rhythm observation (reference treatment strategy) with respect to the primary composite endpoint of fatal and nonfatal events. The occurrence of the composite primary endpoint will be analysed in a statistical comparison of two proportions of (non-)fatal events. Rates of the primary endpoint are calculated as the proportion of patients in whom the primary endpoint has occurred. Rate differences are defined as the rate under the investigational treatment strategy minus that under the reference treatment strategy. The 95% confidence interval for the rate difference is calculated according to the Farrington–Manning method. Noninferiority of the investigational strategy of immediate discharge will be declared if the 95% confidence interval for the rate difference excludes 9%. Use of 95% confidence interval is equivalent to noninferiority testing with a one-sided type I error (α) of 2.5%. The one-sided *P* value for testing noninferiority with a margin of 9% is calculated according to Farrington–Manning method and must < 2.5% for rejection of the null-hypothesis of inferiority.

All of the analyses are carried out in the population of all randomized patients under application of the intention-to-treat principle, which implies that all primary endpoints are counted irrespective of the implementation of the randomized treatment assignment.

The follow-up period for each patient begins on the day of randomization and will be as long and continues for up to 12 months, with the aim of complete data collection. It is expected that the occurrence of the primary endpoint at 24 h will be known for all patients, except for those patients with early discontinuation of follow-up because of full withdrawal of informed consent.

### Participating centres

In total, 11 Dutch medical centres participate in the RISC trial; these are Amsterdam UMC, Amsterdam; Beatrix Hospital, Gorinchem; Diakonessen Hospital, Utrecht; Dijklander Hospitals, Hoorn; Flevo Hospital, Almere; Gelre Hospitals, Apeldoorn; Haaglanden Medical Centre (HMC), The Hague, Leiden University Medical Center (LUMC), Leiden; Maasstad Hospital, Rotterdam; Rijnstate Hospital, Arnhem; and Tergooi Medical Center, Hilversum.

## Summary

Syncope is a common symptom with a broad differential diagnosis, high prevalence, and substantial health care costs. Current guidelines on syncope recommend discharging patients with a low risk of serious cardiovascular events; however, the optimal timing for discharge of low- and intermediate-risk patients is not defined in the guidelines. In current medical practice, low- and intermediate-risk syncope patients are either discharged immediately or discharged after 24 h in-hospital rhythm monitoring (telemetry). We propose a randomized clinical trial comparing immediate discharge (the intervention) with discharge after 24 h rhythm observation (the comparator) in pre-defined syncope patients classified as low or intermediate risk according to the risk features in the ESC guideline on syncope. The RISC trial aims to determine if admission for 24 h in hospital rhythm observation can be safely omitted, potentially reducing health care costs while improving care for syncope patients. This multicentre trial is currently enrolling patients across 10 teaching hospitals throughout the Netherlands.

## Data Availability

The data underlying this article cannot be shared publicly due to protection of patient privacy and confirmation to GDPR standards. Completely anonymized data will be shared on reasonable request to the corresponding author with approval of the medical ethical board.

## Appendix A Follow-up scheme

**Table:**

	**QUESTIONNAIRES**	**DIRECT CONTACT**
**ENROLLMENT**	Study specific questions + SF-12 + SDF + EQ-5D-5L	An vivo
**DAY 1**	EQ-5D-5L	Telephone
**DAY 2**	EQ-5D-5L	—
**DAY 3**	EQ-5D-5L	—
**DAY 4**	EQ-5D-5L	—
**DAY 5**	EQ-5D-5L	—
**DAY 6**	EQ-5D-5L	—
**DAY 7**	EQ-5D-5L + Holter specific questions	Telephone
**END WEEK 2**	EQ-5D-5L	—
**END WEEK 3**	EQ-5D-5L	—
**1 MONTH**	Study specific questions + SF-12 + SDF + EQ-5D-5L + iMCQ + iPCQ	Telephone
**1 YEAR**	Study specific questions + SF-12 + SDF + EQ-5D-5L + iMCQ + iPCQ	Telephone

## Appendix B Study parameters / primary outcomes:

*Supraventricular tachycardia:*

- Any atrial tachycardia (sustained or non-sustained ≥ 3 consecutive complexes)
- Atrio-Ventricular Nodal Re-entry Tachycardia (AVNRT) or Atrioventricular Re-Entry Tachycardia (AVRT)
- Premature Atrial Complex (PAC): >2 doublets or >2 episodes of 3 s duration of bi- or trigemini and/or >5%
- Atrial Fibrillation *de novo*

*Ventricular tachycardia:*

- Any Ventricular Tachycardia (sustained/non sustained)
- Ventricular Fibrillation
- Premature Ventricular Complexes: >2 doublets or >2 episodes of 3 s duration of bi- or trigemini and/or >5%

*Conduction disorders:*

- Asystole >3sec (including conversion pauses)
- New first degree AV block with PQ > 300 msec
- Progression first degree AV block with 15%
- 2nd degree AV block Mobitz type I and II
- 3rd degree AV block
- Any SA block

*Other:*

- (near-)Syncope recurrence
- Unexplained fall with injury
- All cause death
- Cardiovascular death

## Appendix C Registered conduction disturbances

**Table:**

Conduction disorder	Specific definition	Conduction endpoint
Minimal heart rate	As calculated over 10 s intervals	—
Maximal heart rate	As calculated over 10 s intervals	—
Average heart rate	As calculated over the total registration	—
Pause	No ventricular complex within 3000ms	+ (including conversion pauses)
AV-block	New first, second or third degree atrioventricular block	+
2nd degree AV-block	—	+
Complete/ 3rd degree AV-block	—	+
Atrial fibrillation	—	+ (new)
Atrial flutter	—	+ (new
Other supraventricular tachycardia	—	+ (>3 consecutive complexes)
Paroxysmal supraventricular complex (PSVC) burden	—	+ (>5%)
PSVC doublet	2 PSCs’ in sequence	+
Non-sustained ventricular tachycardia	More than 2 ventricular complexes in sequence, shorter than 30 s	+
Ventricular tachycardia	Ventricular complex in sequence longer than 30 s	+
Paroxysmal ventricular complex (PVC) burden	Ventricular complex	+ (>5%)
Ventricular couplet	2 PVCs’ in sequence	+ (>2 episodes)
Ventricular bigeminy	Alternation between 1 sinus-QRS complex and one PVC for more then 2 recurrences	+ (>2 episodes)
Ventricular trigemini	Alternation between 2 sinus-QRS complexes and one PVC for more then 2 recurrences	+ (>2 episodes)
Other beats	All unclassifiable beats	—
Patient events	Patient registered events	+
Bradycardia	Heart rate lower then 50bpm	—
Tachycardia	Heart rate faster then 100bpm	—
